# GT-Repeat Polymorphism in the HO-1 Gene Promoter Is Associated with Risk of Liver Cancer: A Follow-Up Study from Arseniasis-Endemic Areas in Taiwan

**DOI:** 10.3390/jcm10071489

**Published:** 2021-04-03

**Authors:** Meei-Maan Wu, Fang-I Hsieh, Ling-I Hsu, Te-Chang Lee, Hung-Yi Chiou, Chien-Jen Chen

**Affiliations:** 1 Department of Public Health, School of Medicine, College of Medicine, Taipei Medical University, 11031 Taipei, Taiwan; 2 Graduate Institute of Medical Sciences, College of Medicine, Taipei Medical University, 11031 Taipei, Taiwan; 3 Master Program in Applied Molecular Epidemiology, College of Public Health, Taipei Medical University, 11031 Taipei, Taiwan; 4 School of Public Health, College of Public Health, Taipei Medical University, Taipei 11031, Taiwan; hsiehfangi@tmu.edu.tw (F.-I.H.); hychiou@tmu.edu.tw (H.-Y.C.); 5 Department of Research, Taiwan Blood Services Foundation, Taipei 10066, Taiwan; hsu277@blood.org.tw; 6 Institute of Biomedical Sciences, Academia Sinica, Taipei 11529, Taiwan; bmtcl@ibms.sinica.edu.tw; 7 Genomics Research Center, Academia Sinica, Taipei 11529, Taiwan; chencj@gate.sinica.edu.tw

**Keywords:** heme oxygenase-1, genetic polymorphism, liver cancer, cirrhosis, arsenic, epidemiology

## Abstract

The induction of heme oxygenase-1 (HO-1) has been shown to have therapeutic potential in experimental models of hepatitis and liver fibrosis, which are closely related to liver cancer. In humans, HO-1 induction is transcriptionally modulated by the length of a GT-repeat [(GT)n] in the promoter region. We aimed to investigate the effect of HO-1 (GT)n variants on liver cancer in a human population. We determined the HO-1 genotype in 1153 study subjects and examined their association with liver cancer risk during a 15.9-year follow-up. Allelic polymorphisms were classified as short [S, <27 (GT)n] or long [L, ≥27 (GT)n]. Newly developed cancer cases were identified through linkage to the National Cancer Registry of Taiwan. Multivariate Cox regression analysis was used to evaluate the effect of the HO-1 (GT)n variants. Alpha-fetoprotein (AFP) and cirrhosis history were also examined. The S/S genotype was found to be significantly associated with liver cancer risk, compared to the L/S and L/L genotypes. The S/S genotype group also had a higher percentage of subjects with abnormal AFP levels than other groups. There were significant percentages of cirrhosis among groups who carried S-alleles. Our findings indicate that short (GT)n variants in the HO-1 gene may confer susceptibility to rather than protection from liver cirrhosis/cancer.

## 1. Introduction

Heme oxygenase-1 (HO-1) is the rate-limiting enzyme in heme degradation, leading to the generation of biliverdin, carbon monoxide, and ferric iron. Biliverdin is subsequently converted to bilirubin by biliverdin reductase [1]. HO-1 is highly inducible by a variety of stimuli, most of which are associated with the production of inflammation and oxidative stress [2]. Both in vitro and in vivo studies suggest that increased induction of HO-1 in response to various stimuli represents a cytoprotective defense mechanism protecting cells from inflammation- and oxidation-mediated damage [3]. However, other reports also suggest that the cytoprotective effect may be reversed at high levels of HO-1 expression [4,5]. In humans, transcription expression of HO-1 is modulated by the length of a dinucleotide GT-repeat [(GT)n] in the proximal promoter region of the gene after oxidant challenges [6]. Shorter lengths of (GT)n correlate with higher levels of HO-1 gene expression in a dose-dependent manner [7]. Carriers of genetic variants of shorter (GT)n have been associated with less-severe chronic inflammatory diseases, including chronic obstructive pulmonary disease, cardiovascular disease, vascular disease complications, and some types of cancer [8,9].

Previous reports on the relationship between HO-1 (GT)n variants and cancer risk have presented mixed results [9,10]. Carriage of short (GT)n is reported to be associated with lower risks of oral cancer, gastric cancer, prostate cancer, and adenocarcinoma of the lung. However, short (GT)n has also been correlated with higher risks of gastric cancer, pancreatic cancer, melanoma, and squamous cell carcinoma of the lung. In addition, leukemia patients with short (GT)n alleles were reported to have poor prognosis and survival rate after treatment with chemotherapy or radiotherapy [11,12]. Although the exact reasons for these associations are not known, it is suggested that HO-1 may protect cells from abnormal development while still promoting the progression of tumor cells [5]. In other words, the discrepancies may result from differences in cancer stages. However, our previous study that followed cancer-free individuals through a linkage to registry database indicated that the short (GT)n polymorphism (<27 (GT)n), which is related to higher levels of HO-1 induction, may nonetheless increase the risk of development of nonmelanoma skin cancer and lung squamous cell carcinoma, but not lung adenocarcinoma or urinary tract cancer [13]. To the best of our knowledge, the effect of HO-1 (GT)n promoter variants in liver cancer has not been explored.

The pathogenesis of liver cancer is a multi-step process and its progression may result from preexisting chronic hepatitis and liver cirrhosis. Induction of HO-1 has been shown to confer protection in several experimental models of hepatic injury (hepatitis and fibrosis) as well as of hepatocarcinogenesis, irrespective of the underlying cause being of nutritional, alcoholic, or viral origin [14,15,16,17]. In models of nonviral hepatitis in mice, higher activity of HO-1 has been associated with less severe hepatitis and fibrosis by preventing apoptotic liver damage [18]. In a model of transgenic mice for chronic HBV infection, researchers showed that HO-1 may suppress viral replication without inflammation or obvious liver damage [16]. HO-1 also showed pronounced antiviral activity in HCV infection by increasing antiviral interferon response or inhibiting protease activity in vitro [19,20]. The liver injury, if any, seems to result from a secondary recruitment and activation of immune effector cells [16,21]. HO-1 induction was also shown to interrupt progression of nutritional steatohepatitis in both cultured hepatocytes and db/db mice [17]. Overexpression of HO-1 in HepG2 cells has been shown to inhibit cell migration [22]. HO-1 has thus been proposed for therapeutic use against liver injury and complicated outcomes [10,14]. In contrast, reports of studies of patients with short (GT)n polymorphism (related to high HO-1 induction levels) did not support a role of prevention played by HO-1 in hepatitis or fibrosis control [23,24,25]. In view of these contradictory findings, the relationship of HO-1 (GT)n variants and the risk of liver diseases, including chronic hepatitis, cirrhosis, and cancer, remains to be elucidated.

In this study, we analyzed the size of the HO-1 promoter (GT)n in 1153 human study subjects to assess the possible contribution of genetic variation to liver cancer development in a Taiwanese adult population; a retrospective cohort was established and followed for over 20 years [26]. We also compared the frequency of study subjects with hepatitis and cirrhosis among the groups of (GT)n variants in a subsample of the study cohort. The cohort was recruited from two arseniasis-endemic areas in Taiwan [13]. It has been reported that arsenic exposure through drinking well water is associated with a variety of cancer types, including skin, lung, bladder, and probably others [27,28]. The chemical arsenic is a strong inducer of HO-1 expression frequently used in basic experimental studies [2]. The cohort in the endemic areas provides a unique feature of this observational study with natural experiments to study the relationship between HO-1 induction and the risk of liver cancer.

## 2. Materials and Methods

### 2.1. Study Subjects and Baseline Characteristics

Study subjects were derived from two subcohorts in arseniasis-endemic areas in Taiwan, namely the LMN (three alphabets designated for Homei, Fuhsin, and Hsinming villages, respectively) subcohort and the Lanyang subcohort [29]. Epidemiologic follow-up studies of the cohorts began in 1988–1990 and in 1997–1999, respectively. Descriptions of the areas, recruitment of study subjects, and collection of baseline data, including data obtained from questionnaire interviews and health examinations have been detailed previously [13,26,29,30]. Briefly, residents of the areas had been exposed to arsenic through drinking well water until the late 1990s. Bowen’s disease and nonmelanoma skin cancer associated with typical arsenic intoxication have been consistently observed in the study areas [30,31,32]. More recently, studies demonstrated that lung cancer and urothelial cancer were significantly associated with long-term arsenic exposure via drinking water [32,33,34,35,36,37]. However, the relationship between liver cancer and arsenic exposure presents different results depending on study subjects with or without hepatic viral infection [27,32].

The present study included 1153 study subjects who had baseline data available, successful genotyping, and linkage to registry databases (Section 2.2 and Section 2.3 for detailed methods) from the two study subcohorts. The study subjects, representing 67.7% of the combined 692 and 1010 cohort members from each of the respective area, had been followed-up for the incidence of cardiovascular death and a variety of cancers such as cancers of the skin, lung, and urinary tract [13,38]. Previously identified demographic and life style factors in the endemic areas, including cigarette smoking, alcohol consumption, and arsenic exposure, were considered as potential confounding factors. Regular users of cigarette smoking and alcohol consumption were defined at a frequency of at least three days per week for at least half of a year. The index of arsenic exposure for each study subject was calculated as previously defined [13,36].

Liver-related viral infection and injury markers, including HBsAg (a seromarker of hepatitis B virus [HBV] infection), anti-HCV (a seromarker of hepatitis C virus [HCV] infection), glutamic-oaa transaminase (GOT), glutamic-pyruvic transaminase (GPT), and alpha-fetoprotein (AFP), were retrieved from baseline electronic records for the study subjects of the LMN subcohort. The corresponding liver-related data as well as liver cirrhosis history for the study subjects of the Lanyang subcohort were obtained through a linkage to a community-based health examination conducted by the Yilan Public Health Bureau in 2003–2005 [27].

All the subjects gave their informed consent at the time of enrollment for participation and follow-up [13,26,27,29]. This study was performed in accordance with the Declaration of Helsinki, and approved by the institutional review boards of Taipei Medical University (N201807031), and the Genomics Research Center, Academia Sinica (AS-IRB01-08068 and AS-IRB01-11070).

### 2.2. Determination of HO-1 (GT)n Genotype

The number of (GT)n in the HO-1 gene promoter region was determined as described previously [29]. Briefly, the DNA segments of the (GT)n were amplified by polymerase chain reaction (PCR) with paired primers according to a previous report [39]. The PCR products for the size of (GT)n were analyzed as previously described [29]. The number of (GT)n in the HO-1 gene promoter of the study subjects in the present study ranged from 16 to 38, same as shown in our previous study [29]. In both cohorts, 23 and 30 (GT)n were the two most common alleles, which is consistent with the findings from ours and others on Asian populations [7,40]. We therefore selected 27 (GT)n as a cutoff to classify study subjects in the genetic analysis. GT-repeats of <27 were designated as the short (S) allele, and the repeats of ≥27 as the long (L) allele. All the study subjects were accordingly classified as carriers of L/L, L/S, or S/S genotypes. A total of 26 study subjects had missing values in genotype data because of unsuccessful assay and thus were not included in the analysis of this study.

### 2.3. Follow-Up and Ascertainment of Cancer Cases

The primary interest of this study was newly diagnosed incidence of liver cancer (ICD, 9th Revision (ICD-9) code 155.0). Cases of new primary cancer were ascertained through a data linkage to the profiles in the National Cancer Registry of Taiwan. The vital status of each study subject was ascertained using record linkage with the National Death Registration System in Taiwan. These nationwide registry databases were implemented by the government of Taiwan in 1979 and 1968, respectively, and contained information that is accurate, complete, and updated yearly [34,36]. The percentages of pathological confirmation of liver cancer were 46.74% [41]. Follow-up person-years for each study subject were counted from the date of physical examination to the date of cancer diagnosis, date of death, or the end of follow-up (31 December 2013), whichever came earliest.

### 2.4. Statistical Methods

We used ANOVA or chi-square tests to compare the frequency of baseline characteristics among study groups by the HO-1 genotype, where appropriate. Cox proportional hazards regression was used to estimate the hazard ratio (HR) of baseline characteristics in relation to subsequent liver cancer risk. All study variables were categorized into groups, except for age. Body mass index (BMI), total cholesterol level, and triglyceride level was categorized into two groups using the cutoff points of 27 kg/m^2^, 240 mg/dL, and 150 mg/dL, respectively. For Asian populations, 27 kg/m^2^ represent high risk as recommended by the World Health Organization [42]. Arsenic exposure was categorized into three groups, as suggested previously: ≤300, 300–750, and >750 μg/L [29,43].

To examine the association of the HO-1 genotype with subsequent cancer risk, we calculated the HR derived from the Cox regression analyses building on three genetic models: additive model, dominant model, and recessive model. The additive model treated each genotype as a distinct group and used the L/L genotype as the reference group. Trend tests were estimated on integer scores applied to the genotype groups as a continuous term in the regression model. The dominant model compared the carriers of the S-allele (carrying L/S or S/S genotype) with non-carriers (carrying L/L genotype) and the recessive model compared homozygous S-carriers (S/S genotype) with those carrying either 1 or 2 copies of the L-allele (L/S or L/L genotype).

To determine whether there was an association between the HO-1 genotypes and the liver injury markers, we conducted a cross-sectional study. We calculated the prevalence of the markers for each genotype group and compared it among the genotype groups. We used chi-square tests or Fisher’s exact tests to compare the proportion of study subjects with liver injury markers between the genotype groups based on additive, dominant, or recessive genetic models, where appropriate. A study subject having a GOT level >40 U/I was classified as indicating liver injury. The cutoff points for GPT and AFP levels were 40 U/I and 20 ng/mL, respectively. All statistical analyses were performed using SAS 9.4 (SAS Institute, Cary, NC, USA), and a 2-tailed *p* < 0.05 was considered significant.

## 3. Results

### 3.1. Baseline Characteristics and Liver Cancer Incidence by HO-1 (GT)n Genotype

Table 1 shows the baseline characteristics of the study subjects by the HO-1 genotype. The study subjects who carried homozygous S-alleles (S/S genotype) had a higher percentage of no schooling compared to the other two groups. The proportion of cigarette smokers and alcohol drinkers was slightly higher in the subjects carrying the L/S genotype compared with the other groups. However, these differences were not statistically significant.

After a median of 15.9 years of follow-up, a total of 29 new cases of liver cancer were identified; the overall incidence rate of liver cancer was 141.7 per 10^5^ person-years in these study subjects. When the study subjects were classified by HO-1 genotype, a higher risk of liver cancer was observed in the group of S/S genotype as compared to the other groups with L/L or L/S genotype (Table 1). The incidence rate for the three genotype groups was 253.2, 118.4, and 107.7 per 10^5^ person-years, respectively.

As shown in Table 2, after the adjustment of the other baseline variables, age (HR= 1.04, 95% CI 1.00–1.09) and cigarette smoking (HR= 2.68, 95% CI 0.94–7.63; a borderline significance) were found to be the two most important predictors of an increased risk of liver cancer after 15.9 years of follow-up among these study subjects. The other baseline characteristics, including arsenic exposure, were not found to be associated with the risk of liver cancer.

### 3.2. Association of HO-1 (GT)n Genotype with Liver Cancer Risk

After adjusting for age, gender, cigarette smoking, and arsenic exposure, the HO-1 genotype was found to be significantly associated with liver cancer risk among the study subjects based on an additive model (a trend test, HR = 2.11, 95% CI 1.16–3.87) or a recessive model (HR= 3.07, 95% CI 1.35–6.95) (Table 3). The association was not substantially changed after additional adjustments for alcohol consumption and obesity (BMI ≥ 27 kg/m^2^) (Supplementary Table S1), the two potential risk factors for liver cancer reported in literature. Because previous studies of cultured hepatocytes or experimental mice indicated that HO-1 expression may interfere with HBV/HCV infection, we restricted study subjects to those who were HBV or HCV infection positive and examined for the relationship between the HO-1 genotype and liver cancer risk. As shown in Supplementary Table S2, the results of our analysis did not support a protective role of HO-1 L/S or S/S genotype (related to a higher HO-1 expression level) against liver cancer risk. However, we removed study subjects with hepatic viral infection and repeated the regression analysis and found that the S/S genotype group presented a stronger significant association with an increased risk of liver cancer (HR = 4.86, 95% CI 1.55–15.27, Table 3).

### 3.3. Frequency of Liver-Related Injury Markers According to the HO-1 (GT)n Genotype

**Figure 1:**
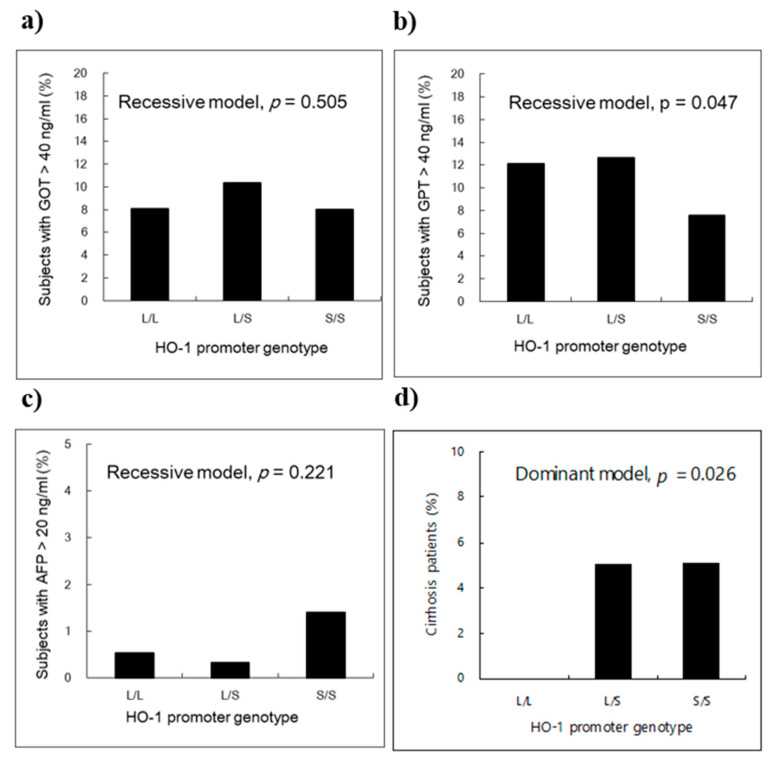
Percentage of study subjects with liver-related injury markers and liver cirrhosis history by the HO-1 promoter genotype. (**a**) Abnormal GOT (>40 U/I in serum). (**b**) Abnormal GPT (>40 U/I in serum). (**c**) Abnormal AFP (>20 ng/mL in serum). (**d**) Liver cirrhosis history. *p*-values were derived fromχ^2^ test based on a dominant or recessive genetic model, where appropriate. The L allele denotes ≥27 GT-repeats and the S allele <27 GT-repeats polymorphism in the HO-1 gene promoter.

Figure 1 shows the percentages of study subjects with abnormal liver-related injury markers according to the HO-1 genotype. The S/S genotype group had a significantly lower percentage of subjects with abnormal GPT levels than the other two groups (*p* = 0.047, Figure 1b); the S/S genotype group had a higher percentage of subjects with abnormal AFP levels, though this difference was not significant (Figure 1c). In addition, there was a significant difference in percentages of liver cirrhosis among the three different HO-1 (GT)n genotype groups, with higher levels in the groups who carried at least one S-alleles (*p* = 0.026, Figure 1d).

## 4. Discussion

In the present study, we examined the association of HO-1 (GT)n variants with liver cancer risk and found that those who carried the S/S genotype had an increased risk of liver cancer during a median 15.9 years of follow-up. We also found that the frequency of individuals with abnormal AFP levels was higher in the group who carried the S/S genotype. Meanwhile, the frequencies of individuals with cirrhosis were significantly higher among individuals with genotypes containing the S-allele. However, the frequency of abnormal GOT or GPT levels was found to be lower in the carriers of the S/S genotype, likely suggesting a tendency to reduce liver inflammation or otherwise an unidentified mechanism. Together, our results indicate that, although the short (GT)n variants in the HO-1 gene promoter may likely be related to a reduced inflammation level, the effect seems not necessarily to confer protection against the development of liver cirrhosis or cancer.

The exact reasons for the discrepancy between mechanism and population studies are not known. In a humanized mice model, Kah et al. reported that HO-1 polymorphisms can affect HCV replication and treatment responses with different efficacy [44]. However, the investigators also suggest that further studies are needed to assess the protective function of HO-1 induction in competent immune systems. HO-1 has been shown to exert anti-inflammatory effects, which may suppress the immune effect of leukocytes in the tumor surveillance of individuals [5], probably after a long-term period of induction [13]. Our present findings, as well as of others [23,24], based on a human population suggest that the effect of HO-1 induction on liver cirrhosis and cancer needs to be examined with caution.

There have been few HO-1 (GT)n studies that directly measured the expression of HO-1 in target tissues and examined their association with either the alleles or human disease [5]. A study report by Taha et al. showed an inverse association of HO-1 levels with the (GT)n allelic variants in primary endothelial cells [45]. The study further showed that the cells carrying shorter alleles survived better under oxidative stress, proliferated more efficiently in response to stimuli, and produced less pro-inflammatory mediators. In our prior study, we also demonstrated an inverse correlation between the (GT)n variants and HO-1 expression levels in skin tissues biopsied from healthy controls or in patients with nonmelanoma skin cancer; with an enhanced expression of HO-1 in the patients [13]. In this study, however, we did not examine the biological effects of HO-1 expression on tissue sections. Hence, the influence of the S-allele on biological effects and thereby on the progression to liver cancer was not known.

Our unexpected findings with reversed results may be explained by environmental factors, such as arsenic exposure, in the study areas. Arsenic, an IARC Class I human carcinogen, is a strong inducer of HO-1 in many cell culture systems [2], which is confirmed in circulating lymphocytes of arsenic-exposed human subjects [46]. The HO-1 induction is thought to act as an adaptive response to oxidant arsenic and functions against oxidative stress in early stages of the exposure. However, the long-term effect of HO-1 has been less well studied. We previously identified specific cancer subtypes that are significantly associated with HO-1 short (GT)n polymorphism (related to high HO-1 induction levels) [13], including Bowen’s disease and nonmelanoma skin cancer and lung squamous cell carcinoma, which are closely related to long-term exposure to arsenic via drinking water. In our prior study, arsenic exposure was shown to increase liver cancer risk in individuals without HBV/HCV infection [27]. In the present study, we examined subjects after excluding those who carry HBV/HCV infection and confirmed that the HO-1 S-allele is still significantly associated with an increased risk of liver cancer after adjusting for arsenic exposure (Table 3). Our data also do not support a role of the S-allele in protection against liver cancer in individuals with viral infection as mechanism studies showed (Supplementary Table S2). We speculate that the long-term exposure to arsenic in the living environment leading to excess HO-1 expression may cause a profound adverse effect on liver damage in individuals with short (GT)n genetic variants.

The correlation between the HO-1 genotype and the cancer risk was significant in additive and recessive models, while the correlation between the HO-1 genotype and liver cirrhosis was only seen in the dominant model. We do not know the exact reason why the short (GT)n is a recessive risk allele (i.e., two alleles are needed) for liver cancer and a dominant risk allele (i.e., one allele is enough) for liver cirrhosis. We speculate that a higher level of HO-1 gene expression might be needed to exert the deleterious effect for cancer development. Further studies with an experimental design are warranted to confirm the above preliminary observations.

The strength of this study is the population-based cohort study design. Previous studies of human subjects regarding HO-1 (GT)n variants are either case-control or cross-sectional study designs, in which differential survival probability may have occurred between groups of various genotypes. The possibility of a biased sample toward less-severe aggressive types cannot be ruled out. By contrast, our study is based on a cohort design with a median of 16 years of follow-up. A reverse causation can therefore be ruled out. HO-1 (GT)n polymorphism in this study is predictive of cancer development, less likely the selection results of progression effect in advanced cancer stages.

There are limitations to this study. First, because of small sample size, we were not able to examine the interaction between arsenic exposure and (GT)n polymorphism on liver cancer risk. Second, although liver cirrhosis is the main risk factor for liver cancer, it was not controlled as a confounding factor in the multivariate Cox regression because of the limited samples obtained from the Lanyang subcohort. Thirdly, as the progression of liver disease is a complex process involving different stages of inflammation, fibrosis, and tumor formation with a wide range of different factors, and there is no analysis in this study based on specific stages of liver disease, whether HO-1 (GT)n is a useful marker in these regards remains unknown. Further studies should be conducted to evaluate whether patients with different stages are associated with short (GT)n variants in the HO-1 gene promoter. Finally, due to the nature of cross-sectional studies, the temporal relationship between HO-1 induction (represented as the (GT)n genotype) and liver cirrhosis, as well as GOT/GPT and AFP, is not clarified in this study. Further studies with follow-up study design are warranted.

## 5. Conclusions

We found that the carriage of short (GT)n variants may increase the risk of liver cirrhosis and cancer. As many research fields of hepatitis propose the therapeutic potential of HO-1 against inflammatory flare in liver damage, our study results may help to allocate patients to a specific risk profile, including the (GT)n genotype before treatment with HO-1 is considered. To support this concept, additional studies with higher numbers of samples in populations of different ethnicities are necessary.

## Figures and Tables

**Table 1:** Baseline characteristics and incidence of liver cancer during follow-up according to HO-1 promoter genotype among 1153 study subjects, 1989–2013.

Characteristics	L/L Genotypes ^1^ (n = 328)	L/S Genotypes (n = 569)	S/S Genotypes (n = 256)	*p*-Value ^3^
Mean age, year	54.9 (11.5) ^2^	56.1 (11.4)	55.7 (10.5)	0.362
Male gender, n	145 (44.2)	262 (46.1)	115 (44.9)	0.861
Education level, n				0.167
No schooling	110 (33.5)	207 (36.4)	111 (43.4)	
Elementary	172 (52.4)	283 (49.8)	111 (43.4)	
Junior high or above	46 (14.0)	78 (13.7)	34 (13.3)	
Cigarette smokers, yes	85 (25.9)	165 (29.0)	67 (26.2)	0.652
Alcohol drinkers, yes	46 (14.0)	92 (16.2)	33 (12.9)	0.728
Body mass index, kg/m^2^				0.370
<27	110 (33.7)	221 (39.3)	94 (37.0)	
23–27	151 (46.3)	229 (40.8)	101 (40.1)	
≥27	65 (19.9)	112 (19.9)	57 (22.6)	
Total cholesterol, mg/dL				0.966
≥240	113 (34.6)	191 (33.7)	86 (34.0)	
Triglyceride, mg/dL				0.202
≥150	98 (30.0)	140 (24.7)	72 (28.5)	
Arsenic exposure, μg/L				0.319
Median (IQR)	357 (76 to 700)	270 (74 to 700)	260 (85 to 700)	
0–50	46 (16.0)	73 (14.6)	25 (12.0)	
50–150	73 (25.4)	141 (28.3)	65 (31.1)	
150–300	15 (5.2)	43 (8.6)	9 (4.3)	
300–750	91 (31.7)	143 (28.7)	64 (30.6)	
>750	62 (21.6)	99 (19.8)	46 (22.0)	
Liver cancer (T155.0) ^4^				
Cases number (n = 29)	7	11	11	
Person-years	5911.5	10,213.3	4344.9	
Incidence rate per 10^5^	118.4	107.7	253.2	

^1^ The L allele denotes ≥27 (GT)n and the S allele <27 (GT)n polymorphism in the HO-1 gene promoter. ^2^ Continuous data are presented as mean (standard deviation) or median (interquartile range (IQR)). Categorical data are given as counts (percentage). The differences between total counts and the total number (n) by genotype are due to missing data. ^3^ ANOVA test for continuous and chi-square test for categorical data. ^4^ ICD-O-FT topographic code. Histological type include 26 cases coded M81703 and 1 coded M80103, and 2 coded M99903.

**Table 2:** Adjusted hazard ratio (HR) of baseline characteristics in relation to liver cancer among the study subjects.

	Age-Gender-Adjusted		Multivariate-Adjusted ^2^	
Characteristics	HR (95% CI)	*p*-Value	HR (95% CI)	*p*-Value
Age, year	1.05 (1.01–1.09)	0.007 ^1^	1.04 (1.00–1.09)	0.049
Gender				
Females	1.00		1.00	
Males	4.23 (1.80–9.91)	0.001	2.78 (0.77–10.0)	0.118
Education level				
No school	1.00		1.00	
Elementary	1.19 (0.49–2.86)	0.704	0.86 (0.33–2.26)	0.759
Cigarette smoking				
No	1.00		1.00	
Yes	1.66 (0.66–4.14)	0.280	2.68 (0.94–7.63)	0.066
Alcohol consumption				
No	1.00		1.00	
Yes	0.47 (0.16–1.41)	0.178	0.51 (0.17–1.58)	0.244
Body mass index, kg/m^2^				
<27	1.00		1.00	
≥27	1.41 (0.60–3.31)	0.438	1.14 (0.41–3.16)	0.805
Total cholesterol, mg/dL				
<240	1.00		1.00	
≥240	0.74 (0.32–1.71)	0.485	0.83 (0.33–2.08)	0.689
Triglyceride, mg/dL				
<150	1.00		1.00	
≥150	1.05 (0.48–2.31)	0.905	1.15 (0.48–2.75)	0.758
Arsenic exposure, μg/L				
≤300	1.00		1.00	
300–750	1.38 (0.53–3.59)	0.505	1.38 (0.52–3.67)	0.515
>750	1.28 (0.43–3.80)	0.660	1.06 (0.33–3.38)	0.923

^1^ Adjusted for gender. ^2^ Adjusted for other variables as listed in the table.

**Table 3:** Multivariate-adjusted hazard ratios (HR) ^1^ of the HO-1 promoter genotype in relation to liver cancer among study subjects.

	All Study Subjects		Subjects without HBV/HCV (+)	
HO-1 Genotype ^2^	HR (95% CI)	*p*-Value	HR (95% CI)	*p*-Value
Additive model				
*L/L*	***1.00***		***1.00***	
L/S	1.36 (0.42–4.37)	0.605	1.06 (0.19–5.84)	0.945
S/S	3.78 (1.18–12.13)	0.025	5.06 (1.01–25.42)	0.049
*Trend test*	2.11 (1.16–3.87)	0.015	2.79 (1.14–6.82)	0.025
Dominant model				
L/L	1.00		1.00	
L/S or S/S	2.01 (0.68–5.39)	0.204	2.02 (0.44–9.27)	0.367
Recessive model				
L/L or L/S	1.00		1.00	
S/S	3.07 (1.35–6.95)	0.007	4.86 (1.55–15.27)	0.007

^1^ Adjusted for age, gender, cigarette smoking, and arsenic exposure. CI: confidence interval. ^2^ The L allele denotes ≥27 GT-repeats and the S allele <27 GT-repeats polymorphism in the HO-1 gene promoter.

## Data Availability

The datasets of the National Cancer Registry and Death Registry System analyzed and/or generated during this study are held by the Ministry of Health and Welfare (MOHW) of Taiwan. The data are available from the corresponding author with the permission of the Taiwan MOHW.

## Supplementary Materials

The following are available online at https://www.mdpi.com/article/10.3390/jcm10071489/s1, Table S1: Multivariate-adjusted hazard ratios (HR) of HO-1 promoter genotype in relation to liver cancer among study subjects, Table S2: Multivariate-adjusted hazard ratios (HR) of HO-1 promoter genotype in relation to liver cancer among study subjects with HBV/HCV infection.
