# Supplementary material for: GT-Repeat Polymorphism in the HO-1 Gene Promoter Is Associated with Risk of Liver Cancer: A Follow-Up Study from Arseniasis-Endemic Areas in Taiwan
Source: J Clin Med. 2021 Apr 3;10(7):1489. doi: 10.3390/jcm10071489 (PMC8038349; doi:10.3390/jcm10071489)
Supplement: Supplementary file 1 [file jcm-10-01489-s001.pdf]

Supplementary Materials

Supplementary Table S1. Multivariate-adjusted hazard ratios (HR) [1] of HO-1 promoter genotype in relation to liver cancer among study subjects.

	**All Study Subjects**		Without HBV/HCV (+)	
**HO-1 Genotype** [2]	**HR (95% CI)**	*p*-Value	HR (95% CI)	*p*-Value
Additive model				
L/L	1.00		1.00	
L/S	1.37 (0.43-4.41)	0.594	1.09 (0.20-5.96)	0.925
S/S	3.68 (1.15-11.82)	0.028	5.14 (1.03-28.82)	0.047
Trend test	2.07 (1.14-3.78)	0.018	2.79 (1.14-6.82)	0.024
Dominant model				
L/L	1.00		1.00	
L/S or S/S	2.01 (0.68- 5.90)	0.205	2.06 (0.45- 9.44)	0.353
Recessive model				
L/L or L/S	1.00		1.00	
S/S	2.97 (1.31- 6.74)	0.009	4.88 (1.55-15.33)	0.007

[1] Adjusted for age, gender, cigarette smoking, alcohol consumption, BMI, and arsenic exposure. CI: confidence interval.

[2] The L allele denotes ≥ 27 GT-repeats and the S allele < 27 GT-repeats polymorphism in the HO-1 gene promoter.

Supplementary Table S2. Multivariate-adjusted hazard ratios (HR) of HO-1 promoter genotype in relation to liver cancer among study subjects with HBV/HCV infection.

**HO-1 Genotype**	**Model I** [1]		Model II [2]	
	**HR (95% CI)**	*p*-Value	HR (95% CI)	*p*-Value
Additive model				
L/L	1.00		1.00	
L/S	1.55 (0.30-7.92)	0.598	1.42 (0.27-7.33)	0.678
S/S	1.69 (0.30-9.48)	0.549	1.69 (0.30-9.51)	0.554
Trend test	1.27 (0.57-2.84)	0.566	1.28 (0.56-2.93)	0.557
Dominant model				
L/L	1.00		1.00	
L/S or S/S	1.61 (0.34- 7.50)	0.547	1.52 (0.32- 7.15)	0.597
Recessive model				
L/L or L/S	1.00		1.00	
S/S	1.25 (0.36- 4.27)	0.725	1.32 (0.38- 4.58)	0.660

[1] Model I: adjusted for age, gender, cigarette smoking, and arsenic exposure. CI: confidence interval.

[2] Model II: additionally, adjusted for alcohol consumption and BMI. BMI: body mass index.
